# A Preliminary Study on the Biased Attention and Interpretation in the Recognition of Face-Body Compound of the Individuals with Social Anxiety

**DOI:** 10.3389/fpsyg.2016.00414

**Published:** 2016-03-24

**Authors:** Dong-Hyun Kim, Jang-Han Lee

**Affiliations:** Clinical Neuro-psychology Lab, Department of Psychology, Chung-Ang UniversitySeoul, South Korea

**Keywords:** social anxiety, attention bias, interpretation bias, face-body compound, eye-movement

## Abstract

The present study aimed to investigate the processes through which individuals with social anxiety (SA) attend to and interpret compound emotional expressions of the face and body. Incongruent face-body compound images that combined an angry face (or body) with a fearful, sad, or happy body (or face) were presented to a SA group (*n* = 22) and a healthy control (HC) group (*n* = 22). The participants were instructed to interpret the emotional state of the image, and their eye-movements and behavioral responses were measured. The results revealed that both group showed longer scanpath length during the recognition of compound images which combined angry face with angry, fearful, sadness, or happy body. The SA group also showed longer scanpath length in congruent face-body compound images of fear and sad. Additionally, the SA group fixated for a shorter period of time on the face and longer on the body than the HC group. Regarding emotion interpretation, the SA group was more likely to interpret the emotional state of incongruent face-body compound images based on the body than the HC group. These findings provide a preliminary observation that individuals with SA showed different attentional bias pattern by congruency of face-body compound images and that it might have biased their interpretations of the emotional states.

## Introduction

Social anxiety (SA) disorder is defined as a persistent fear of social situations in which an individual is exposed to unfamiliar people or to possible scrutiny by others (American Psychiatric Association, 1994). According to cognitive bias theory (Clark and Wells, 1995), fear about the evaluation of others would produce biased information processing of the potential social threat. This attentional bias would lead to interpretation of social situations more negatively (Blanchette and Richards, 2010) and thus contributes to maintenance of SA (Clark and Wells, 1995).

Attentional bias is comprised of two sequential processes: automatic and strategic (Cisler and Koster, 2010). When a social cue is presented, socially anxious individuals show a hypervigilance behavioral pattern toward the cue automatically to search for potential threat cues. However, after perceiving the cue, they tried to avoid it. This attentional avoidance (i.e., the strategic process mentioned above) is an important characteristic of SA that reflects emotion regulation strategy to alleviate negative emotion.

This hypervigilance-avoidance pattern has been investigated using several experimental set ups using isolated faces as stimuli and adopting eye-movement measurement (Staugaard, 2010). The most popular experimental set up is presenting more than two social cues per trial and measuring where, how long, and how often participants fixated among presented social cues. It had been revealed that socially anxious individuals showed more first fixations to negative facial expression and relatively lower fixation time for negative (e.g., angry, disgust, or fear) facial expression compared to healthy controls when staring simultaneously at neutral and emotional face pairs (Wieser et al., 2009).

On the other hand, for an experimental set up presenting one isolated face per trial, different approach was required. Horley et al. (2004) found that SA patients showed a greater total scanpath length for angry faces, compared to healthy individuals. They also showed overall reduced fixation count and dwell time of the eyes. This avoidance of eye contact was especially greater when confronted with an angry face.

Although previous studies of hypervigilance-avoidance provided useful insights into SA, these studies are limited in that they focused on limited experimental set ups using isolated facial images. Given that negative facial expression can be a salient sign of disapproval, rejection, and hostility (Staugaard, 2010), the human face offers useful social cues for research on SA. However, in trivial life, individuals perceive facial expressions that are not isolated but occur in combination with other contextual information, such as voice, bodily expressions, and social situation (For review, see Gilboa-Schechtman and Shachar-Lavie, 2013). Specifically, the human body provides very important social cues in conjunction with the face (Aviezer et al., 2012). Recent neuroimage studies have even found that the neural mechanism of body perception is very similar to that of face perception (Hadjikhani and de Gelder, 2003; Meeren et al., 2005). Therefore, it is necessary to investigate the attentional bias of the individuals with SA using integrated, combined faces and bodies.

This integrative processing of the face and body has been investigated by the face-body compound paradigm, which manipulate the ambiguity by presenting incongruent facial and bodily emotion expression compounds (Peschard et al., 2014). Regarding the categorization of the facial and bodily expression of these images, researchers have observed Stroop-like interference to miscategorize the emotion of bodily expressions as facial expressions (Meeren et al., 2005; Aviezer et al., 2008; Mondloch, 2012) and vice versa (Kret, 2011).

Interestingly, the intensity of Stroop-like interference was modulated by emotion pair type of face-body compound. Aviezer et al. (2008) found that participants showed relatively stronger stroop like interference in disgust face and anger body compound images than that of disgust face and fear body. This may due to the similarity between the emotion conveyed by the face and that of body. According to the circumplex model of affect (Russell, 1997), facial expressions are not directly categorized into specific emotions, but convey the values on the orthotomic grid with two continuous dimensions: arousal and valence (whether the target’s emotional state is pleasant or unpleasant). In this perspective, emotional similarity represents the distance between coordinates on this affective grid. In other word, high similarity between incongruent facial and bodily expression causes strong ambiguity, and thus causes more intensive Stroop-like interference effect.

In addition, Shields et al. (2012) observed a facial bias, which involves greater fixation on the face than the body and an associated interpretation of the emotional state according to the emotion perceived in the face (∼75% of trials) rather than that in the body (∼25% of trials) during the interpretation of the overall emotional statement of the incongruent face-body compounds. The average body fixation time of the trials in which participants choose the emotion shown in the body was relatively longer than that of the trials in which participants choose the emotion shown in the face. In contrast, the average face fixation time of the trials in which participants choose the emotion shown in the body was relatively longer than that of the trials in which choose the emotion shown in the face.

Aviezer et al. (2012) proposed the Gestalt-like unit model, in which the face and body are not distinct components, but a holistic single configuration. It is similar with human face processing, which perceives facial components as an integrated holistic single configuration. Facial bias in face-body compound recognition may be due to the human face being the most salient cue in face-body compound perception, just as the dominant component in facial processing are individual features such as eye, nose, and mouth (Ekman et al., 2001). Stroop-like interference is also explainable by confusion during the holistic process of face and body information in the early stage of emotion perception.

The present study sought to extend the research scope of attentional bias involving SA from isolated face to face-body compound images. In detail, we investigated the hypervigilance-avoidance pattern and its influence on emotion interpretation by measuring eye-movement and behavioral response during recognition of face-body compounded images. Based on Gestalt-like unit model (Aviezer et al., 2012), we assumed that attentional process involving the perception of face-body compounds would be similar to that of isolated face. Following the studies on isolated face perception (Horley et al., 2004; Moukheiber et al., 2010), hypervigilance was defined as increased total scanpath length, and avoidance was defined as reduced fixation ratio on the face.

Since anger expression is a salient cue for the individuals with SA (Staugaard, 2010), it was used in this study as a target emotion. Given that emotional similarity between facial and bodily expression modulates the intensity of Stroop-like interference (Aviezer et al., 2008), we add three other emotions based on distance from anger in terms of coordinates of the circumplex model (Russell, 1997): fear (high similarity with anger), sadness (medium similarity with anger), and happiness (low similarity with anger). We created congruent images that combined face and body with the same emotion. Incongruent images that combined anger expressions in the face (or body) with expressions of happiness, sadness, or fear in the body (or face) were also made. The participants were instructed to view the image of the face-body compound and select the emotional state that best described the image.

## Materials and Methods

### Participants

Seven-hundred-twenty-one undergraduate students of the Chung-Ang University were screened using the Korean version of the Social Avoidance and Distress Scale (SADS; Watson and Friend, 1969) and the Brief Version of the Fear of Negative Evaluation 2 (BFNE2; Carleton et al., 2006). Based on the criteria of Lee and Choi (1997), prior to the initiation of the experiments, we defined the SA group (*N* = 22, age *M* = 22.05, *SD* = 2.82; 11 male) as those who scored above 99 on the SADS and below the highest 20% on the BFNE2 and the Healthy Control (HC) group (*N* = 22, age *M* = 20.64, *SD* = 2.17; 11 male) as those who scored below 63 on the SADS and above the lowest 20% on the BFNE2. We prospectively included all participants who signed an informed consent that had been approved by the institutional review board (Chung-Ang Psychology Research Ethics Committee).

### Measures

The SADS consists of 28 items for measuring the avoidance of social situations, and social interaction anxiety (e.g., I try to avoid situations which force me to be very sociable). Each item was rated on a 5-point scale (1 = not at all; 5 = very much so). The total score ranges from 28 to 140, with a higher score indicating greater social avoidance and distress. The internal consistency in the current sample was Chronbach’s *α* = 0.98.

The BFNE2 consists of 11 items for measuring nervousness in evaluative condition and social approval (e.g., I am frequently afraid of other people noticing my shortcomings). Each item is rated on a 5-point scale (1 = not at all characteristic of me; 5 = extremely characteristic of me). The scores range from 11 to 55, with a higher score representing greater fear of negative evaluation. The internal consistency in the current sample was Chronbach’s *α* = 0.97.

The State-Trait Anxiety Inventory (STAI; Spielberger et al., 1970) consists of 20 trait version (STAI-T) items for measuring a general, long-term form of anxiety (e.g., I worry too much over something that really doesn’t matter), and 20 state version (STAI-S) items for measuring a temporary form of anxiety (e.g., I am tense). Each item is rated on a 4-point scale (1 = not at all; 4 = very much). Both STAI-T and STAI-S have scores ranging from 20 to 80, with a higher score indicating greater trait and state anxiety, respectively. The internal consistencies of STAI-T and STAI-S were Chronbach’s *α* = 0.94 and 0.92, respectively.

### Materials and Apparatus

The face and body images were obtained from the Korea University Facial Expression Collection (KUFEC; Lee et al., 2006), Yonsei facial expression database (Bahn et al., 1997), Japanese Female Facial Expressions (JAFFE; Lyons et al., 1998), Chung-Ang Emotional Bodily Expression Stimuli (CEBES; Kim and Lee, 2013), and Bodily Expressive Action Stimulus Test (BEAST; de Gelder and Van den Stock, 2011).

A pilot study was conducted to select the stimulus images. Twenty graduate students were asked to select the emotion that they perceived in each of the images from among the following four emotions: happiness, anger, sadness, and fear. They were also asked to rate the perceived emotional intensity of each image on a 7-point Likert scale (1 = no intensity; 7 = high intensity). The emotion selection accuracy and mean intensity were calculated. Twenty images (eight angry, four sadness, four fearful, and four happy) that elicited the highest accuracy rates were selected for each face and body. The accuracy rates and emotional intensities of the selected images are presented in **Table 1**.

**Table 1:** Mean (SD) accuracies and emotional intensity ratings for the face and body images.

	Face		Body	
		
	Accuracy rate	Emotional intensity	Accuracy rate	Emotional intensity
Anger	96.43 (5.40)	3.97 (1.32)	94.64 (7.39)	4.02 (1.26)
Happy	100.00 (0.00)	4.09 (1.45)	96.43 (6.84)	4.43 (1.24)
Sadness	96.43 (4.12)	4.13 (1.32)	100.00 (0.00)	4.71 (1.14)
Fearful	80.36 (3.57)	3.88 (1.51)	98.21 (3.57)	4.82 (1.28)


**FIGURE 1:**
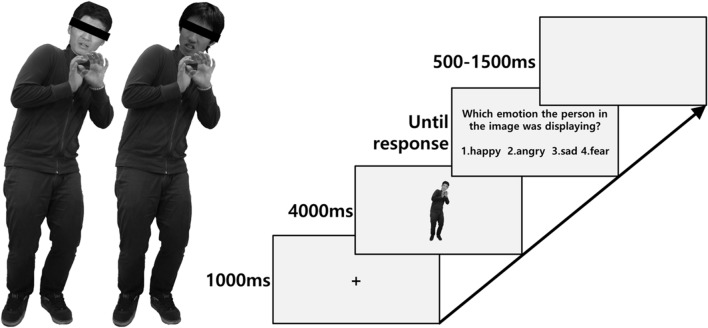
**Example of the congruent (**left**; fearful face on fearful body), incongruent (**middle**; anger face on fear body) face-body compound image and **(right)** outline of an experimental trial.** Due to portrait rights issue, we masked eyes of example images.

We created two different types of face-body compounds: congruent and incongruent compounds (see **Figure 1**). The congruent compounds consisted of faces and bodies with the same emotion (e.g., an angry face on angry body). The incongruent compound consisted of faces and bodies with different emotions (e.g., a happy face on an angry body). Six types of incongruent compounds were created: angry faces on fearful bodies, fearful faces on angry bodies, angry faces on sad bodies, sad faces on angry bodies, angry faces on happy bodies, and happy faces on angry bodies.

In the compounding process, each face and body image was used twice (e.g., an angry face was combined once with an angry body and once with a happy body). A total of 16 congruent compound images and 24 incongruent compound images (see **Table 2**) were made. The height of the images was 18 cm (16.7°). The images were presented on a Dell P2210 monitor (22-inch, WSXGA, 1680 × 1050 pixels). The iViewX RED-IV system (SensoMotoric Instruments, Teltow, Germany) was used to record eye movements and key responses.

**Table 2:** The number of images in congruent face-body compound condition (A) and incongruent face-body compound condition (B).

(A) Congruent face-body compound images	Emotion			
	
	Anger	Fear	Sad	happy
Male	2	2	2	2
Female	2	2	2	2

**(B) Incongruent face-body compound images**	**Modality of anger**	**Emotion compound type**		
		
		**Anger–fear**	**Anger–sad**	**Anger–happy**

Female	Face	2	2	2
	Body	2	2	2
Male	Face	2	2	2
	Body	2	2	2


### Procedures

Before the start the experiment, the participants sat in a chair in a shielded room and completed the STAI. Next, they were asked to place their head on a chinrest at a distance of 60 cm from the monitor. The eye-tracker was calibrated for each participant. Subsequently, the experimental session, which consisted of two practice trials and 40 experimental trials, was conducted. Each trial began with the appearance of a fixation cross for 1,000 ms (ms). Then, the stimulus image was presented for 4,000 ms. After the image disappeared, an instruction to select the emotional state that best described the individual in the image by pushing a keyboard button (1 = happy, 2 = sadness, 3 = anger, 4 = fearful) was presented. Finally, to reduce the monotonous nature of the task, a white screen was presented for a variable time interval between 500 and 1,500 ms (see **Figure 1**). The trials were presented in random order. The entire experimental session took approximately 20 min, and the participants were debriefed and provided with a monetary reward (∼5 US dollars).

### Data Analysis

The data from the trials in which the selected emotion did not match that of the face or body were excluded (e.g., the trial with anger face and sad body which was categorized as fear; 10% of the data). Due to a technical malfunction, the data from 5 participants were excluded. Finally, the data from 39 participants (20 SA, 19 HC) were used in the analyses. The statistical analyses were performed with SPSS 17.0 (SPSS Inc., Chicago, IL, USA).

For the eye-movement analysis, we measured total scanpath length and fixation time on the face and body. Total scanpath length was defined as the cumulative distance in pixel between sequential fixation points. For the congruent compound trials, two-way repeated-measures analysis of variances (ANOVA) was conducted on total scanpath length with emotion (anger, fear, sadness, happiness) as a within-group variable and group (SA and HC) as a between-group variable. For the incongruent compound trials, three-way repeated-measures ANOVAs were conducted on the total scanpath length with emotion compound type (anger–fear, anger–sadness, and anger–happiness) and anger modality (face and body) as within-group variables and group (SA and HC) as a between-group variable.

**FIGURE 2:**
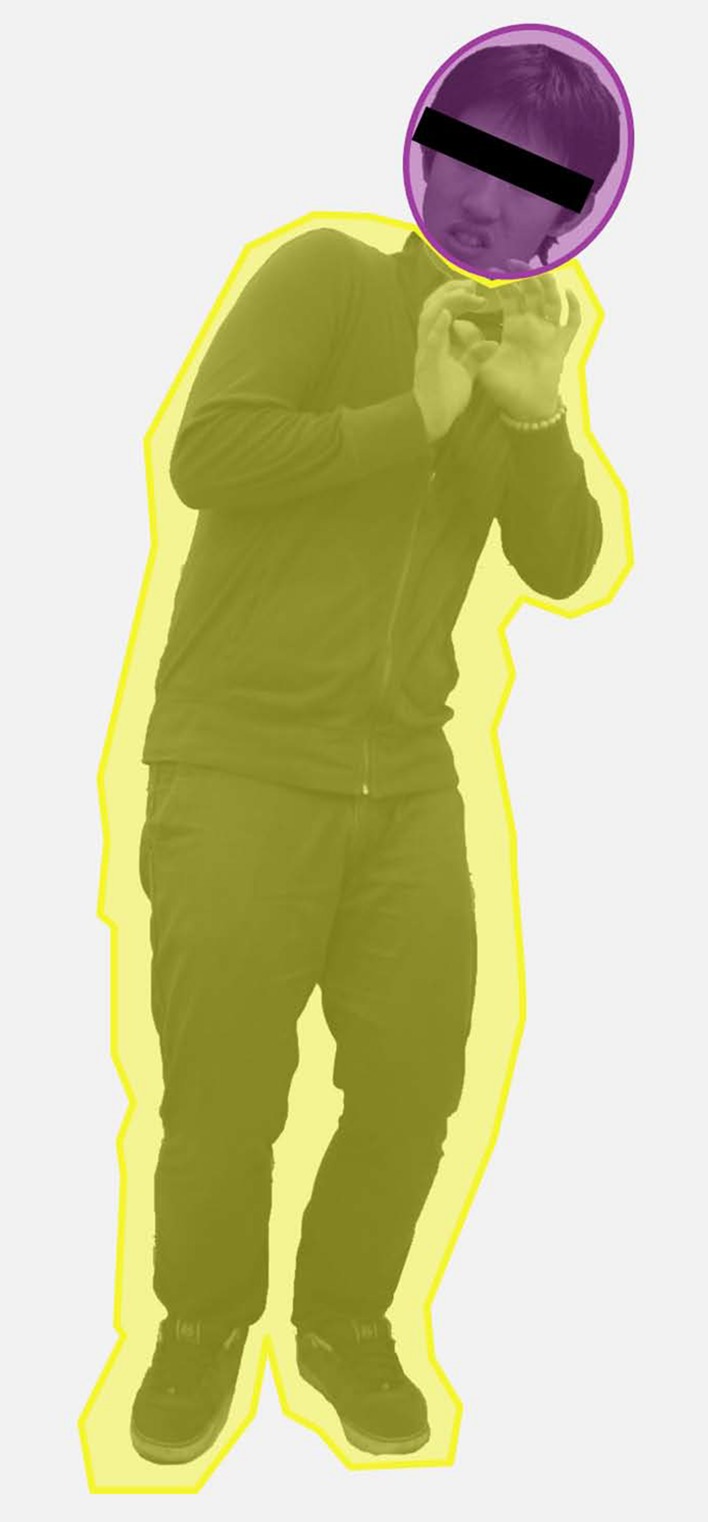
**Representive polygon shape of AOI over the face and the body.** Due to portrait rights issue, we masked eyes of example images.

To investigate where the participants preferred to fixate (i.e., on the face or body), we set area of interests (AOIs) over the faces as ovals that covered the face (see **Figure 2**). Because an oval- or square-shaped of AOI cannot properly cover the dynamic pose of a body, we drew a polygon shape based on the pose of the body in the image to set the AOI of the body. We calculated a facial attention bias score (FABS) by dividing the fixation time on AOI over the face by total fixation time on the AOI over the face and the body. A FABS above 0.5 reflected an attention bias toward the face, and a FABS below 0.5 reflected n attention bias toward the body. For the congruent compound trials, two-way repeated-measures ANOVA was conducted on FABS with emotion (anger, fear, sadness, happiness) as a within-group variable and group (SA and HC) as a between-group variable. For the incongruent compound trials, three-way repeated-measures ANOVAs were conducted on FABS with emotion compound type (anger–fear, anger–sadness, and anger–happiness) and anger modality (face and body) as within-group variables and group (SA and HC) as a between-group variable.

For the analyses of the behavioral measurements, we investigated the participants’ tendencies to interpret incongruent compound images as expressing anger by calculating an anger interpretation bias score (AIBS) as follows: the number of anger interpretation responses was divided by the total number of responses. AIBSs above 0.5 reflected a tendency to interpret the images as expressing anger, and AIBSs below 0.5 reflected a tendency to interpret the images as expressing emotions other than anger. A three-way repeated-measures ANOVAs were conducted on the AIBS with emotion compound type (anger–fear, anger–sadness, and anger–happiness) and anger modality (face and body) as within-group variables and group (SA and HC) as a between-group variable. Follow-up *t*-tests were conducted to investigate the simple effects of the groups and emotion compound types on the AIBSs. In addition, one sample *t*-test was conducted on AIBS, to investigate whether the percentage of anger interpretation was significantly higher or lower than the chance level (50%).

## Results

### Descriptive Measures

**Table 3** shows the demographic information for the SA and HC groups. The two groups did not significantly differ in age [*t*(37) = -1.82, *p* > 0.05] or gender [χ*^2^* = 0.23, *df* = 1, *p* > 0.05]. The SA group exhibited significantly higher scores on the SADS [*t*(37) = 14.45, *p* < 0.01], BFNE2 [*t*(37) = 9.49, *p* < 0.01], STAI-T [*t*(37) = 8.04, *p* < 0.01], and STAI-S [*t*(37) = 7.03, *p* < 0.01] than the HC group. It is indicated that participants in both group did not differ in basic demographic factors except the degree of SA level.

**Table 3:** Mean (SD) for demographics information.

Variable	Social anxiety group (*n* = 20)	Healthy control group (*n* = 19)	*t*
Gender (% male)	55.00%	47.36%	
Age	20.65 (2.13)	22 (2.49)	-1.82
SADS	105.45 (4.71)	59.68 (13.33)	14.45ˆ*
BFNE2	44.65 (3.87)	25.37 (8.18)	9.49ˆ*
STAI-T	51.6 (7)	36.68 (4.15)	8.04ˆ*
STAI-S	46.15 (8.27)	31.53 (3.81)	7.03ˆ*


*^∗^*p* < 0.01; SADS, Social Avoidance and Distress Scale; BFNE2, Brief version of Fear of Negative Evaluation 2; STAI-T, State-Trait Anxiety Inventory-Trait, STAI-S, State-Trait Anxiety Inventory-State.*

### Eye-Movement Measures

For the total scanpath length of congruent compounds, the main effect of emotion [*F*(3,111) = 5.17, *p* < 0.005, η^2^ = 0.123] and the interaction between emotion and group [*F*(3,111) = 3.54, *p* < 0.05, η^2^ = 0.087] was significant. For follow-up analysis on each group, we conducted paired sample *t*-test on four emotions with Bonferroni-corrected significance level of 0.0083 to control familywise multiple comparison issue. HCs showed relatively greater total scanpath length in anger congruent images than that of happy [*t*(18) = 2.96, *p* < 0.0083] congruent images. The SA group showed relatively greater total scanpath length in happy congruent images than that of anger [*t*(19) = -3.60, *p* < 0.0083], fear [*t*(19) = -4.49, *p* < 0.0083] than that of happy congruent images.

To sum up, the HC group showed relatively greater total scanpath length in anger-congruent images than in happiness-congruent images. The SA group showed relatively greater total scanpath lengths in anger-, and fear-congruent images than that of happiness-congruent images. The greater total scanpath lengths indicated that HCs may show a hypervigilance pattern on anger-congruent compounds only, while SAs showed this pattern on not only the congruent compounds of anger, but also that of fear.

**FIGURE 3:**
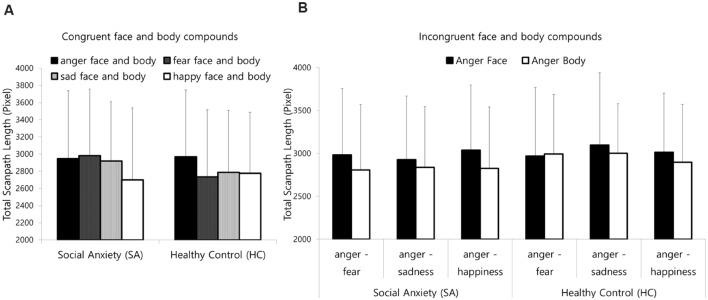
**Mean total scanpath length for **(A)** the congruent face and body compounds and **(B)** incongruent compounds.** Error bars represent standard deviation of the means.

For the total scanpath length of incongruent compounds, the main effect of modality was significant [*F*(1,37) = 6.63, *p* < 0.05, η^2^ = 0.152]. As presented in **Figure 3**, both group showed overall greater scanpath length when modality of anger was face than that of anger body. Other main effects and interactions were not significant. In other word, both group showed hypervigilance pattern when anger face is presented with bodily expression of fear, sadness, or happy.

**FIGURE 4:**
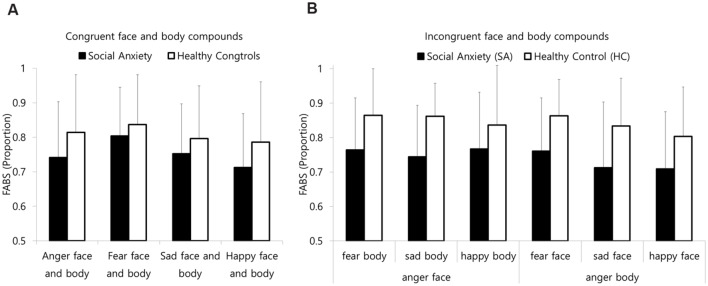
**Mean FABS for **(A)** the congruent compounds and **(B)** the incongruent compounds FABS: facial attention bias score.** A FABS above 0.5 reflects an attentional bias toward the face. Error bars represent standard deviation of the means.

The results of three-way repeated-measures ANOVA on the FABS of incongruent compound images revealed a main effect of group [*F*(1,37) = 147.23, *p* < 0.05, η^2^ = 0.147]. Other main effects and interactions were not significant. As presented in **Figure 4**, the FABS for the SA group was relatively lower than that of the HC group. In other words, the SA group showed avoidance toward face that spending less time fixating on the face and more time fixating on the body than the HC group, regardless of the emotion compound type or the anger modality.

Regarding the congruent compound images, the results of two-way repeated-measures ANOVA on the congruent compound images revealed that the group difference was not significant [*F*(1,37) = 1.68, *p* = 0.20, η^2^ = 0.043]. The main effect of the FABS of emotion was significant [*F*(3,111) = 3.19, *p* < 0.01, η^2^ = 0.102]. The FABS for the fear compound images was marginally higher than that for the sadness [*t*(38) = 1.99, *p* = 0.054] and significantly higher than that for the happy [*t*(38) = 3.16, *p* < 0.05] compound images.

### Behavioral Measures

The results of three-way repeated-measures ANOVA on the AIBS revealed significant interactions between the emotion compound type and the anger modality [*F*(2,74) = 152.27, *p* < 0.01, η^2^ = 0.81], emotion compound type and group [*F*(1,37) = 3.73, *p* < 0.05, η^2^ = 0.09], and anger modality and group [*F*(1,37) = 8.56, *p* < 0.05, η^2^ = 0.12]. To follow-up on these two-way interactions, separate two-way ANOVAs for emotion compound type and group were conducted on each anger modality.

**FIGURE 5:**
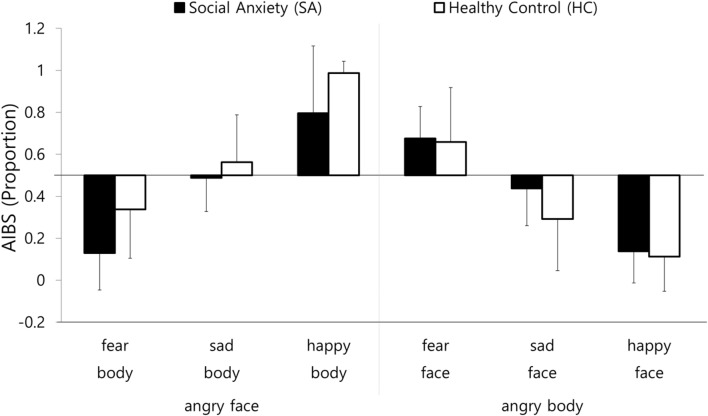
**Mean AIBS for the anger face and anger body condition.** AIBS: Anger Interpretational Bias Score. An AIBS above 0.5 reflects a tendency to interpret the image as expressing anger, and an AIBS below 0.5 reflects a tendency to interpret the image as expressing an emotion other than anger. Error bars represent standard deviation of the means.

As presented in **Figure 5**, when the anger modality was the face, the AIBS of the SA group was significantly lower than that of the HA group [*F*(1,37) = 9.58, *p* < 0.01, η^2^ = 0.21]. These findings suggest that the SA group was less likely to interpret the emotional state of the angry face and other emotional bodily expression compounds as anger compared to the HC group. Apart from the group difference, the main effect of the emotion compound type was also significant [*F*(2,74) = 85.49, *p* < 0.01, η^2^ = 0.70]. A follow-up analysis revealed that the AIBS for the angry face on the fearful body was significantly lower than the AIBSs for the angry face on the sad body [*t*(38) = -6.65, *p* < 0.01] and the angry face on the happy body [*t*(38) = -12.73, *p* < 0.01]. The AIBS for the angry face on the sad body was significantly lower than that for the angry face on the happy body [*t*(38) = -6.66, *p* < 0.01].

When the anger modality was the body, the interaction between the emotion compound type and group was marginally significant [*F*(2,74) = 3.10, *p* = 0.051, η^2^ = 0.08]. A follow-up analysis revealed that the AIBS of the SA group was significantly higher than that of the HA group for the fearful face on the angry body [*t*(37) = 2.50, *p* < 0.05]. However, for the fearful face on the angry body and the happy face on the angry body, there were no significant main effects of group (see **Figure 4**). These findings indicate that the SA group interpreted the emotional state of the fearful face on the angry body as anger to a greater extent than the HC group. In other words, the SA group was more likely to interpret the emotional state of the fearful face on the angry body compound image based on the body than the HC group.

Apart from the interaction between group and emotion compound type, the main effect of the emotion compound type was significant [*F*(2,74) = 77.10, *p* < 0.01, η^2^ = 0.68]. A follow-up analysis revealed that the AIBS for the fearful face on the angry body was significantly higher than the AIBS for the sad face on the angry body [*t*(38) = 5.83, *p* < 0.01] and the AIBS for the happy face on the angry body [*t*(38) = 12.58, *p* < 0.01]. The AIBS for the sad face on the angry body was significantly higher than that of the happy face on the angry body [*t*(38) = 6.05, *p* < 0.01].

In order to follow-up the main effect of the emotion compound type in both groups, one-sample *t*-tests were conducted on the AIBS. The results are presented in **Table 4**. For the anger–fear compound, the AIBS of angry face-fear body was significantly higher than the chance level. In contrast, the AIBS of fear face-angry body was significantly lower than the chance level. In other words, they interpret the emotional state of the angry-fearful compound based on the body.

**Table 4:** Mean (SD) for AIBS of emotion compound type, modality of anger, and group.

Group	Modality of anger	Angry–fearful compound		Angry–sad compound		Angry–happy compound	
				
		AIBS	*t*	AIBS	*t*	AIBS	*t*
SA^a^	Face	15.42 (20.82)	-7.43ˆ**	51.25 (17.99)	0.31	79.58 (32.04)	4.13ˆ**
	Body	65 (17.85)	3.76ˆ**	47.5 (21.13)	-0.53	12.5 (15.17)	-11.05ˆ**
HC^b^	Face	35.53 (22.54)	-2.8ˆ*	58.77 (21.24)	1.80	95.18 (16.03)	12.28ˆ**
	Body	65.35 (26.54)	2.52ˆ*	28.95 (25.21)	-3.64ˆ**	11.84 (16.74)	-9.94ˆ**


*^∗^*p* < 0.05; ^∗∗^*p* < 0.01; ^a^*df* = 19; ^b^*df* = 18; SA, social anxiety; HC, healthy control; AIBS, anger interpretation bias score.*

For the anger-sadness compound, the AIBS of both modality of anger was not significantly different from the overall chance level. Exceptionally, the AIBS of sad face on angry body was significantly lower than the chance level. For the angry-happy compound, the AIBS in the angry face condition was significantly higher than the chance level. In contrast, the AIBS in the angry body condition was significantly lower than the chance level.

In other words, anger-sad compound based on angry-happy compound based on the face. To sum up, these indicated that participants interpreted the emotional state in a different way by the emotion compound type.

## Discussion

The individuals with SA in present study showed a complex pattern in attention and interpretation process by the level of SA and the congruency of face-body compound images. SA group revealed hypervigilance pattern without avoidance toward the face for congruent face-body compounded images. On the contrary, they showed avoidance toward the face without hypervigilance. Based on previous studies on attentional bias that the hypervigilance and avoidance mediated by different neural mechanisms (automatic process and strategic process; Cisler and Koster, 2010), we propose a preliminary model that the activation of automatic and strategic process is closely related with ambiguity of stimuli, which is modulated by congruency of face-body compounds.

In this model, the HC group showed a hypervigilance pattern without avoidance toward face in face-body compound images with anger face, regardless of bodily expression of emotion. This is because an angry face could be a potential threat (Staugaard, 2010) and thus, activates an automatic process. However, since face-body compound images are not actually threatening, they did not activate a strategic process.

On the other hand, the SA group showed vigilance toward congruent face-body compound images of not only anger, but also fear and sadness. This may be due to SA group’s high anxiety level (see **Table 2**), and consequent lower threshold for activation of automatic process. Interestingly, they did not show avoidance toward faces in congruent face-body compound image. This may due to stimuli features. In previous studies on hypervigilance pattern on isolated face perceptions, isolated face images were presented in huge size (e.g., Horley et al., 2004 = 14.9°; Moukheiber et al., 2010 = 26.57°). This may cause hypervigilance of individuals with SA by inducing a threatening feeling of somebody staring at participants at close range. Comparing with isolated face, face in the face-body compound image was presented in a relatively small size. It induced a subjective feeling that the face-body compound image is located at relatively greater distance, thereby bypassing activation of strategic process.

For incongruent face-body compound images, the SA group only showed avoidance toward face without hypervigilance. This may be due to the interaction between ambiguity of incongruent compound images and the potential threat of an angry face. The ambiguity of incongruent compound images causes bias in interpretation of face-body compound images causes toward interpreting social cues in a more negative way. They thereby trigger the strategic process regardless of subjective perception of distance.

The behavioral measurements revealed that the SA group was more likely to interpret the emotional state based on the body than the HC group. In accordance with the results of the eye-movement measures, these results suggested that the individuals with SA exhibited a tendency to avoid the face (Horley et al., 2004; Moukheiber et al., 2010) and were thus relatively more concerned with the body while interpreting the emotional states of other people.

To elaborate this preliminary model, several methodological issues should be fixed in future research. Firstly, the present study adopted a relatively indirect way of measuring average of total scanpath length and fixation time during a long presentation time (4,000 ms). In this design, we cannot completely exclude alternative explanations. For example, the relatively longer fixation time on body exhibited by the SA group could be interpreted not as avoidance toward face, but as a difficulty in disengagement from the body.

Secondly, more rigid control for the stimuli image set is required. In present study, isolated face and body images with three negative emotions (anger, fear, sadness) and one positive emotion (happiness) were used. Furthermore, every incongruent image consisted of angry face (or body) in conjunction with other bodily expressions. In this condition, we cannot rule out the possibility that these compounded images can lead any biased interpretation or habituation. In addition, even though we selected isolated face images that staring at the front, some of the face stimuli have slightly averted eye gaze. Given that averted gaze enhanced perception of avoidance-oriented emotions such as fear and sadness (Adams and Kleck, 2005), it is required to strictly control the eye gaze direction for precise measurement of the effect of ambiguity in incongruent compound perception. The absence of neutral face and neutral body compounds as control stimuli also weaken the validity of the hypervigilance- avoidance hypothesis. In future research, more direct measurements such as first fixation, and fixation time in separated time interval, more strict stimuli selection, and various types of face-body compounds should be adopted.

Regarding the sample size, the participants in the present study were university students, and the sample size was small (20 SAs, 19 HCs). The severity of SA in the SA group may have been relatively lower than that of individuals who are clinically diagnosed with SA disorder. Moreover, the small sample size might have altered the effect sizes (Kühberger et al., 2014) and thus reduced the statistical validity. Given these points, the present study should be replicated with larger clinical samples.

Nonetheless, this finding provides a preliminary exploration of the process of interpreting of social cues in individuals with SA. Previous studies of interpretation bias have suggested that individuals with SA tend to interpret ambiguous social cues in a negative way (Blanchette and Richards, 2010). However, in the present study, the SA group may have strategically avoided gazing at the face and were thus more concerned with the bodily gestures regardless of whether the emotional valence of the face or body was positive or negative. If this assumption is valid, the individuals with SA interpreted the facial expression less negatively and interpreted the bodily expression more negatively than the healthy individuals. This facial avoidance could be temporarily helpful for alleviating negative emotions during interactions with individuals who are expressing negative emotions on their faces. In contrast, this avoidance could cause miscommunications and malfunctions in social communication by inducing excessive concern with bodily gestures and thus aid the maintenance of SA by causing misinterpretations of the emotional states of other people.

Regardless of the attentional bias and its influence on emotion interpretation, the present study also found the relationship between the intensity of Stroop-like interference and emotional similarity between emotions conveyed in face and body. Basically, the face in the face-body compound is the most salient cue for emotion interpretation, as is the case with the facial components (e.g., eyes, nose, and mouth) in isolated face (Aviezer et al., 2012). However, as presented in **Figure 4** and **Table 4**, the participants interpreted emotional state in different ways by the emotion compound type. They tended to interpret the emotional state of the angry–happy compound through facial expression. For the angry–happy compounds, the likelihood of interpreting the emotion through the facial expression did not significantly differ with the chance level (50%). For the angry–fearful compound, they tended to interpret the emotional state through the bodily gestures rather than facial expression. This pattern is consistent with the findings of Aviezer et al. (2008). That is, as the emotional similarity between face and body is greater, the intensity of Stroop-like interference by the bodily expression being miscategorized as facial expression is also greater.

In summary, the present study provided some preliminary findings that indicated that individuals with SA may strategically avoid the face, and this attentional pattern could affect the interpretation of the emotional state. Given the results of the present study, it is necessary to extend the focus of cognitive bias research from isolated faces to face-body compounds.

## Author Contributions

All of the authors have contributed in this study with design, data collection, and interpretation of the results. The manuscript was drafted by D-HK with supervision of J-HL. All authors have read and corrected draft versions, and approved the final version.

## Conflict of Interest Statement

The authors declare that the research was conducted in the absence of any commercial or financial relationships that could be construed as a potential conflict of interest.
